# Anti-Müllerian hormone: correlation with testosterone and oligo- or amenorrhoea in female adolescence in a population-based cohort study

**DOI:** 10.1093/humrep/deu182

**Published:** 2014-07-23

**Authors:** P. Pinola, L.C. Morin-Papunen, A. Bloigu, K. Puukka, A. Ruokonen, M.-R. Järvelin, S. Franks, J.S. Tapanainen, H. Lashen

**Affiliations:** 1 Department of Obstetricsand Gynaecology, Oulu University Hospital, University of Oulu and Medical Research Center Oulu, Oulu, Finland; 2 Department of Children and Young People and Families, National Institute for Health and Welfare, Aapistie 1, Box 310, FI-90101 Oulu, Finland; 3 NordLab Oulu, Oulu University Hospital and Department of Clinical Chemistry, University of Oulu, Oulu, Finland; 4 Department of Epidemiology and Biostatistics, MRC Health Protection Agency (HPA) Centre for Environment and Health, School of Public Health, Imperial College London, London, UK; 5 Institute of Health Sciences, University of Oulu, PO Box 5000, FI-90014 Oulu, Finland; 6 Biocenter Oulu, University of Oulu, Aapistie 5A, PO Box 5000, FI-90014 Oulu, Finland; 7 Unit of Primary Care, Oulu University Hospital, Kajaanintie 50, PO Box 20, FI-90220 Oulu, 90029 OYS, Finland; 8 Institute of Reproductive and Developmental Biology, Imperial College London, London, UK; 9 Department of Obstetrics and Gynaecology, University of Helsinki and Helsinki University Central Hospital, Helsinki, Finland; 10 Department of Human Metabolism, University of Sheffield, Jessop Wing, Sheffield, UK

**Keywords:** AMH, female adolescence, PCOS, oligo- or amenorrhoea, testosterone

## Abstract

**STUDY QUESTIONS:**

Can serum anti-Müllerian hormone (AMH) levels measured in female adolescents predict polycystic ovary syndrome (PCOS)-associated features in adolescence and early adulthood?

**SUMMARY ANSWER:**

AMH levels associated well with PCOS-associated features (such as testosterone levels and oligoamenorrhoea) in adolescence, but was not an ideal marker to predict PCOS-associated features in early adulthood.

**WHAT IS KNOWN ALREADY:**

Several studies have reported that there is a strong correlation between antral follicle count and serum AMH levels and that women with PCOS/PCO have significantly higher serum AMH levels than women with normal ovaries. Other studies have reported an association between AMH serum levels and hyperandrogenism in adolescence, but none has prospectively assessed AMH as a risk predictor for developing features of PCOS during adulthood.

**STUDY DESIGN, SIZE, DURATION:**

A subset of 400 girls was selected from the prospective population-based Northern Finland Birth Cohort 1986 (*n* = 4567 at age 16 and *n* = 4503 at age 26). The population has been followed from 1986 to the present.

**PARTICIPANTS/MATERIAL, SETTING, METHODS:**

At age 16, 400 girls (100 from each testosterone quartile: 50 with oligo- or amenorrhoea and 50 with a normal menstrual cycle) were selected at random from the cohort for AMH measurement. Metabolic parameters were also assessed at age 16 in all participants. Postal questionnaires enquired about oligo- or amenorrhoea, hirsutism, contraceptive use and reproductive health at ages 16 and 26.

**MAIN RESULTS AND ROLE OF CHANCE:**

There was a significant correlation between AMH and testosterone at age 16 (*r* = 0.36, *P* < 0.001). AMH levels at age 16 were significantly higher among girls with oligo- or amenorrhoea compared with girls with normal menstrual cycles (35.9 pmol/l [95% CI: 33.2;38.6] versus 27.7 pmol/l [95% CI: 25.0;30.4], *P* < 0.001). AMH at age 16 was higher in girls who developed hirsutism at age 26 compared with the non-hirsute group (31.4 pmol/l [95% CI 27.1;36.5] versus 25.8 pmol/l [95% CI 23.3;28.6], *P* = 0.036). AMH at age 16 was also higher in women with PCOS at age 26 compared with the non-PCOS subjects (38.1 pmol/l [95% CI 29.1;48.4] versus 30.2 pmol/l [95% CI 27.9;32.4], *P* = 0.044). The sensitivity and specificity of the AMH (cut-off 22.5 pmol/l) for predicting PCOS at age 26 was 85.7 and 37.5%, respectively. The addition of testosterone did not significantly improve the accuracy of the test. There was no significant correlation between AMH levels and metabolic indices at age 16.

**IMPLICATIONS, REASONS FOR CAUTION:**

AMH is related to oligo- or amenorrhoea in adolescence, but it is not a good marker for metabolic factors. The relatively low rate of participation in the questionnaire at age 26 may also have affected the results. AMH was measured in a subset of the whole cohort. AMH measurement is lacking international standardization and therefore the concentrations and cut-off points are method dependent.

**WIDER IMPLICATIONS FOR THE FINDINGS:**

Using a high enough cut-off value of AMH to predict which adolescents are likely to develop PCOS in adulthood could help to manage the condition from an early age due to a good sensitivity. However, because of its low specificity, it is not an ideal diagnostic marker, and its routine use in clinical practice cannot, at present, be recommended.

**STUDY FUNDINGS AND COMPETING INTERESTS:**

The study was funded by a grant from Wellcome Trust (089549/Z/09/Z) to H.L., S.F. and M.-R.J. Study funding was also received from Oulu University Hospital Research Funds, Sigrid Juselius Foundation and the Academy of Finland. None of the authors have any competing interest to declare.

## Introduction

Anti-Müllerian hormone (AMH) plays a central role in sexual differentiation by inducing the regression of the Müllerian ducts in male fetuses. In females, AMH is produced in the granulosa cells of the human ovary after mid-gestation (Vigier *et al.*, 1984; Rajpert-De Meyts *et al.*, 1999; Kuiri-Hänninen *et al.*, 2011). AMH is expressed in granulosa cells of growing follicles up to the antral stage, suggesting an important role in early ovarian folliculogenesis (Weenen *et al.*, 2004; Stubbs *et al.*, 2005). AMH is able to inhibit the initiation of primordial follicle growth (Durlinger *et al.*, 2002) and may also decrease the sensitivity of antral follicles to follicle-stimulating hormone (FSH) (Durlinger *et al.*, 1999, 2002; Gruijters *et al.*, 2003). Testosterone has been shown to lower AMH expression in the mammalian ovary *in vitro* (Crisosto *et al.*, 2009). In humans, however, the association between androgens and AMH remains uncertain, and its exact function in follicular recruitment and long-term effects is not well understood.

Polycystic ovary syndrome (PCOS) is the most common endocrine disorder in women, producing symptoms of hyperandrogenism, oligo- or amenorrhoea and polycystic ovaries (PCO) (Franks 1995; Ehrmann 2005). Serum AMH levels and the ovarian antral follicle count (AFC) correlate closely both in healthy subjects and in women with PCOS (Pigny *et al.*, 2003, 2006; Weenen *et al.*, 2004). In PCOS, serum AMH correlates positively with serum concentrations of testosterone (T) and negatively with age (Piltonen *et al.*, 2005). Furthermore, women with higher serum levels of AMH and T have longer menstrual cycles compared with those with lower levels (Kristensen *et al.*, 2012). In line with these observations, in non-hirsute girls with oligo-amenorrhoea (OA), the levels of AMH are similar to those in the PCOS population but higher than in girls with normal cycles (Hart *et al.*, 2010a,b; Park *et al.*, 2010a,b). AMH has been reported to be not associated with metabolic risks in adolescence and in early adulthood (Lin *et al.*, 2011; Anderson *et al.*, 2013), but contradictory results have been published in adult female populations (Nardo *et al.*, 2009; Park *et al.*, 2010a,b; Skalba *et al.*, 2011).

The use of the measurement of serum AMH serum levels as a diagnostic tool for PCOS has been recently under debate (Dewailly *et al.*, 2011; Eilertsen *et al.*, 2012; Iliodromiti *et al.*, 2013). Women with PCOS have higher concentrations of AMH, and accordingly, AMH correlates with the AFC. Thus, AMH has been proposed to be a substitute for AFC in the diagnosis of PCOS (Pigny *et al.*, 2006; Dewailly *et al.*, 2011; Eilertsen *et al.*, 2012). AMH has also been reported to correlate with other symptoms of PCOS, such as hyperandrogenism and oligoamenorrhoea (Pigny *et al.*, 2003; Laven *et al.*, 2004; Piltonen *et al.*, 2005; Nardo *et al.*, 2009; Li *et al.*, 2011; Skalba *et al.*, 2011). There are, however, no follow-up studies on the subject of AMH as a possible predictor of PCOS and its typical symptoms (oligo- or amenorrhoea, hirsutism and hyperandrogenism) in later life.

PCOS is also associated with increased metabolic risks in later life (Legro 2002; Solomon *et al.*, 2002; Nisenblat and Norman 2009). For clinicians to be able to prevent adverse health events in PCOS, predicting the syndrome early in life is important. We have recently reported that oligo- or amenorrhoea at the time of adolescence is associated with hyperandrogenaemia and may represent a risk factor for the development of PCOS in adulthood. Furthermore, we have also reported an association between obesity, hyperandrogenaemia and metabolic risks among 16-year-old adolescent girls (Pinola *et al.*, 2012). We hypothesize that AMH could be used as an early marker for reproductive and metabolic future risks linked to PCOS to allow early preventive actions such as lifestyle changes.

The main aim of the present study was to elucidate the relationship between serum AMH and testosterone levels, oligo- or amenorrhoea, and metabolic and cardiovascular markers at the age of 16, and to evaluate whether AMH can be used as a marker for predicting future cycle irregularities, hirsutism and diagnosis of PCOS.

## Materials and Methods

### Study Population

The study population was a subset of 400 subjects, from the Northern Finland Birth Cohort 1986 (NFBC-86), selected to be assayed for serum AMH.

The prospective NFBC-86, comprised 9362 mothers and their 9479 births (9432 children born alive), who had an expected date of birth between 1 July 1985 and 30 June 1986, drawn from the northernmost part of Finland. In 2001–2002, when the children were 16 years old, the adolescents and their parents received postal questionnaires. Of the female adolescents (*n* = 4567) then living in Finland, either in the original catchment area or elsewhere; 80.3% answered the questionnaire and 74% underwent clinical examination (i.e. anthropometric measurements) and gave fasting blood samples. After excluding twins and triplets, pregnant girls (*n* = 20), oral contraceptive users and users of other forms of hormonal contraception and treatment (*n* = 377), and subjects with incomplete data (*n* = 824), 2448 singleton females remained eligible.

The selection of the study population for the measurement for serum AMH was completed among the subjects who answered both questionnaires at ages 16 and 26. The study population was split into testosterone quartiles and the final group of study subjects (100 subjects from each testosterone quartile: 50 subjects with reported oligo- or amenorrhoea and 50 with normal menstrual cycles) were randomly selected from each quartile using a validated statistical method (SPSS software).

The questionnaire included a question about the regularity and length of the menstrual cycle: ‘Is your menstrual cycle (the interval from the beginning of one menstrual period to the beginning of the next period) often (more than twice a year) longer than 35 days?’. In addition, the girls answered to the following question: ‘If you are using oral contraceptives, how long was your menstrual cycle during the year before you started any oral contraceptives?’. The girls who had a menstrual cycle often (more than twice a year) longer than 35 days at the time of the questionnaire and/or before the use of oral contraceptives were considered to be suffering from oligo- or amenorrhoea.

In 2012, when the subjects were 26 years old, a new questionnaire enquiring about the menstrual cycles was posted to all the girls who participated in the previous survey at age 16. This questionnaire included the same question on oligo- or amenorrhoea as at age 16. Further, the incidence of hirsutism was also self-assessed using a modified Ferriman and Gallwey (F&G) score sheet. The total response rate for 26-year-old questionnaire was 50.4%. The subjects of the subpopulation used in the present study had answered both questionnaires. The diagnosis of PCOS was enquired from the questionnaire (answer to the question: ‘Have you been diagnosed for PCOS by a physician?’). Additionally, women with both hirsutism and oligo- or amenorrhoea according to the questionnaire at age 26 were considered to have PCOS according to both the NIH and the Rotterdam criteria (Rotterdam ESHRE/ASRM-Sponsored PCOS consensus workshop group, 2004).

The Ethics Committee of the Northern Ostrobothnia Hospital District approved this study and informed consent was obtained from all subjects.

### Assays

At age 16, serum samples were assayed for testosterone (T) using Agilent triple quadrupole 6410 LC/MS equipment with an electrospray ionization source operating in positive-ion mode (Agilent Technologies, Wilmington, DE, USA). Multiple reaction monitoring was used to quantify testosterone by using d3-testosterone. The intra-assay CVs of the method were 5.3, 1.6 and 1.2% for testosterone at 0.6, 6.6 and 27.7 nmol/l, respectively. The inter-assay CVs were 5.3, 4.2 and 1.0% for the respective concentrations. In an adult female population, an upper limit of 2.3 nmol/l is considered as the upper normal limit in our laboratory.

Plasma glucose, total cholesterol, high-density lipoprotein cholesterol (HDL-cholesterol), low-density lipoprotein cholesterol (LDL-cholesterol) and triglycerides, fasting insulin, high sensitivity C-reactive protein (hsCRP) and sex hormone binding globulin were all assayed as previously described (Pinola *et al.*, 2012). To quantify the degree of insulin sensitivity, homeostasis model assessment (HOMA) values were calculated using the validated calculator available at http://www.dtu.ox.ac.uk.

Serum AMH concentration was assayed in the stored samples by AMH Gen II enzyme linked immunosorbent assay (ELISA, Beckman Coulter, Inc., 250 S. Kraemer Blvd. Brea, CA 92821 USA) as previously described (Kumar *et al.*, 2010; Wallace *et al.*, 2011). Intra- and inter-assay coefficients of variation were 4.6 and 8.0% at 0.05 ng/ml concentration; 1 ng/ml of AMH converts to 7.14 pmol/l.

### Data analysis

The study population of 400 girls was constituted from four equal groups (quartiles) according to their testosterone levels. The AMH levels were compared between the four quartiles. In the whole study population and within each testosterone quartile, a correlation analysis was carried out between AMH and incidence of irregular anovulatory cycles/oligo-amenorrhea and hirsutism based on the F&G scores obtained at age 26. Further, in the whole population and in each testosterone quartile AMH was correlated with metabolic and cardiovascular risk markers (waist–hip ratio, serum fasting insulin, plasma fasting glucose, total cholesterol, HDL-cholesterol, LDL-cholesterol, triglycerides, hsCRP and HOMA for insulin sensitivity), adjusting for testosterone and BMI.

### Statistical methods

The distribution of AMH was skewed; therefore it was logarithmically transformed to achieve normality in the analyses of hirsutism. The Chi square test was used to compare categorical variables between the testosterone quartiles. The confidence intervals for proportions were calculated using CIA computer program (Gardner and Altman 1989). The potential trends in continuous variables across the testosterone quartiles were examined using the test for linear trend available in analysis of variance (ANOVA). In order to be able to adjust for confounding variables, ANOVA and analysis of covariance (ANCOVA) were adopted when comparing AMH levels between two groups. Correlations were tested using Pearson's correlation analysis. Partial correlation analysis was used to control for confounding variables. Bias-corrected and accelerated 95% confidence intervals of the correlation coefficients were calculated with 1000 bootstrap resamples. We constructed a receiver operating characteristic (ROC) curve with AMH and testosterone at age 16 for PCOS at age 26. The cut-off value of AMH and testosterone were selected to identify PCOS subjects, with the best sensitivity and specificity. Analyses were performed by using SPSS 18.0 software (SPSS, Inc., Chicago, IL, USA).

## Results

The background characteristics of the study population are shown in the Table I. At age 16, the BMI was significantly increased towards greater testosterone quartile (*P*-value for trend <0.001). Mean levels of testosterone in testosterone quartiles were 1.05 in the first, 1.46 in the second, 1.83 in the third and 2.49 nmol/l in the fourth quartile.

**Table I:** Background characteristics of the study population.

Characteristics of study population		Whole population	Testosterone quartile				*P*-value^e^
			1st	2nd	3rd	4th	
Socio-economic state 16 years^a^ (*N* = 370)	White collar	21.9% (17.7; 26.1)	17.7% (10.7; 26.8)	22.6% (14.6; 32.4)	15.6% (8.8; 24.7)	31.9% (22.5; 42.5)	0.126
	Blue collar	49.2% (44.1; 54.3)	53.1% (42.7; 63.4)	43.0 % (32.8; 53.7)	52.2% (41.4; 62.9)	48.4% (37.7; 59.1)	
	Worker	17.0% (13.2; 20.9)	17.7% (10.7; 26.8)	17.2% (10.2; 26.4)	18.9% (11.4; 28.5)	14.3% (7.8; 23.2)	
	Other	11.9% (8.6; 15.2)	11.5% (5.9; 19.6)	17.2% (10.2; 26.4)	13.3% (7.1; 22.1)	5.5% (1.8; 12.4)	
Alcohol consumption^b^ 16 years (*N* = 399)		20.3% (16.4, 24.2)	20.0% (12.7; 29.2)	22.2% (14.5; 31.7)	15.0% (8.7; 23.5)	24.0% (16.0; 33.6)	0.421
Smoking^c^ 16 years (*N* = 399)		13.3% (9.5, 16.6)	12.0% (6.4; 20.0)	15.0% (8.7; 23.5)	9.1% (4.2; 16.6)	17.0% (10.2; 25.8)	0.375
Age at menarche^d^ (year, *N* = 320)		13.1 (1.1)	13.4 (1.0)	12.9 (1.0)	13.0 (1.2)	13.0 (1.0)	0.059
BMI 16 years^d^ (kg/m^2^, *N* = 399)		21.0 (2.9)	20.3 (2.4)	21.0 (2.9)	21.2 (2.9)	21.7 (3.2)	<0.001
BMI 26 years^d^ (kg/m^2^, *N* = 342)		24.2 (7.5)	23.7 (4.7)	24.5 (5.2)	23.5 (3.7)	25.0 (12.8)	0.455
WHR 16 years^d^ (*N* = 398)		0.77 (0.04)	0.77 (0.04)	0.77 (0.04)	0.77 (0.05)	0.77 (0.04)	0.781
WHR 26 years^d^ (*N* = 302)		0.84 (0.08)	0.84 (0.08)	0.85 (0.10)	0.85 (0.08)	0.83 (0.08)	0.573

BMI, body mass index; WHR, waist–hip ratio.

^a^Socio-economic state according to subjects (age 16) mothers, frequency reported, 95% confidence interval in parentheses.

^b^Once per month or more, frequency reported, 95% confidence interval in parentheses.

^c^2–4 days per week or more, frequency reported, 95% confidence interval in parentheses.

^d^Mean of the variable reported, standard deviation in parentheses.

^e^*P*-value for linear trend of continuous variables and for difference between quartiles of category variables. One-way ANOVA for continuous variables and *χ*^2^-test for category variables.

### AMH and testosterone

**Figure 1:**
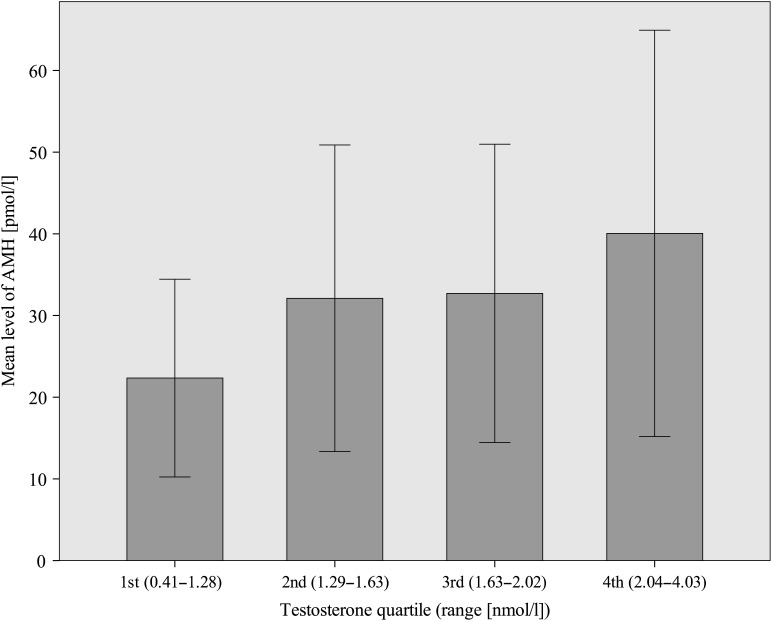
Mean anti-Müllerian hormone (AMH) levels in different testosterone (T) quartiles. Standard deviations of the means are shown in the bars. *P*-value <0.001 for trend from the lowest towards the highest T quartile.

There was a significant correlation between serum AMH and T at age 16 [*r* = 0.36, 95% confidence interval (95% CI) 0.24–0.47, *P* < 0.001]. After dividing the study population into T quartiles, the correlation was significant only in the highest T quartile (*r* = 0.33, 95% CI 0.01–0.56, *P* = 0.001). These results were independent of BMI, WHR and age at menarche. AMH levels increased significantly from the lowest towards the highest T quartile (*P*-value for trend <0.001, Fig. 1).

### AMH and oligo- or amenorrhoea

**Figure 2:**
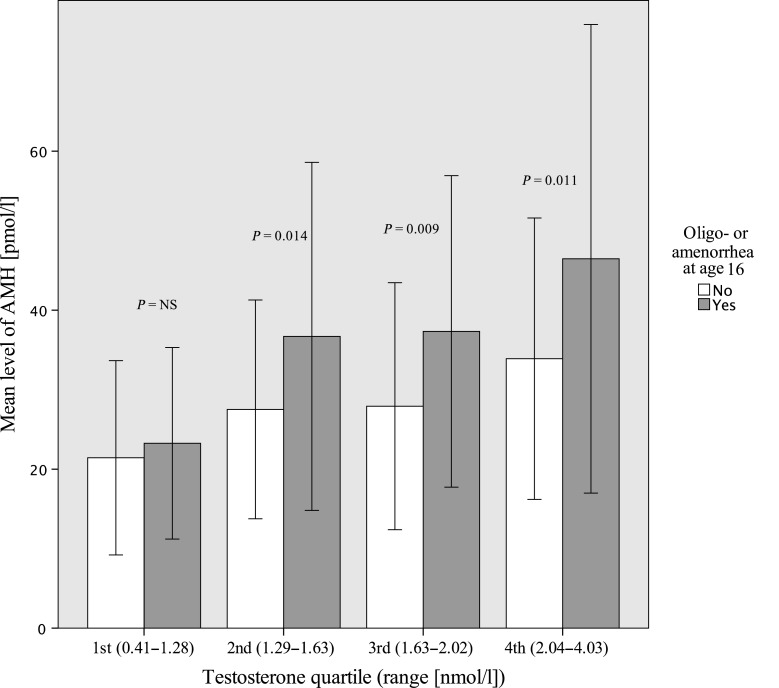
Mean anti-Müllerian hormone (AMH) levels, in the subjects with oligo- or amenorrhoea (grey) and in the subjects with normal menstrual cycles (white), in testosterone quartiles, at age 16. Standard deviations of the means are shown in the bars. *P*-value for the difference between girls with oligo- or amenorrhoea and girls with normal menstrual cycle in each T quartile.

At age 16, the girls who reported oligo- or amenorrhoea (*n* = 200) had higher AMH levels compared with those reporting normal cycles (*n* = 200) [35.9 pmol/l (95% CI: 33.2; 38.6) versus 27.7 pmol/l (95% CI: 25.0; 30.45), *P* < 0.001], and adjustment for BMI, WHR or T did not change the results. Subjects with oligo- or amenorrhoea had higher serum AMH levels in the second, third and fourth T quartiles compared with those with regular menstrual cycles (Fig. 2). Serum AMH levels at age 16 did not differ significantly between women with oligo- or amenorrhoea and women without oligo- or amenorrhoea at age 26. However, within the highest testosterone quartile, the women with oligo- or amenorrhoea (*n* = 23) at age 26 had higher AMH levels at age 16, compared with women with normal menstrual cycles (*n* = 62) at age 26, after adjusting for T and BMI [45.5 pmol/l (95% CI: 36.0; 55.1) versus 36.1 pmol/l (95% CI: 30.3; 41.9), *P* = 0.04].

### AMH and hirsutism

After exclusion of oral contraceptive users (*n* = 122), women with evidence of hirsutism at age 26 (based on self-reported F&G score >7) had significantly higher AMH levels at age 16 compared with non-hirsute counterparts (Table II). However, in the whole population, including the oral contraceptive users, at age 26 the statistically significant difference disappeared. Similarly, the difference disappeared after exclusion of all those using any hormonal contraceptives (oral contraceptive pill, minipill, hormone intrauterine device, subcutaneous implant or injection) at age 26.

**Table II:** Anti-Müllerian hormone (AMH) concentrations at age 16 in women with hirsutism or polycystic ovary syndrome (PCOS) at age 26.

		Mean AMH concentration (pmol/l)^a^	*P*-value
Hirsutism at age 26^b^ (Ferriman & Gallwey score > 7)^c^	Yes (*n* = 34) 13.2 (11.8; 14.6)^d^	31.4 (27.1; 36.5)^e^	0.036
	No (*n* = 171) 4.4 (3.0; 5.9)^d^	25.8 (23.3; 28.6)^e^	
PCOS at age 26^f^	Yes (*n* = 21)	38.2 (27.8; 48.5)	0.044
	No (*n* = 209)	30.2 (27.9; 32.5)	

^a^95% confidence interval in parentheses.

^b^According to modified Ferriman & Gallway score (>7).

^c^73 subjects missing from the analysis due to the non-response to some items, 122 oral contraceptive users excluded.

^d^Mean Ferriman & Gallway score, 95% confidence interval in parentheses.

^e^Geometric mean for AMH.

^f^170 subjects missing from the analysis due to the non-response to some items.

### AMH and PCOS

**Figure 3:**
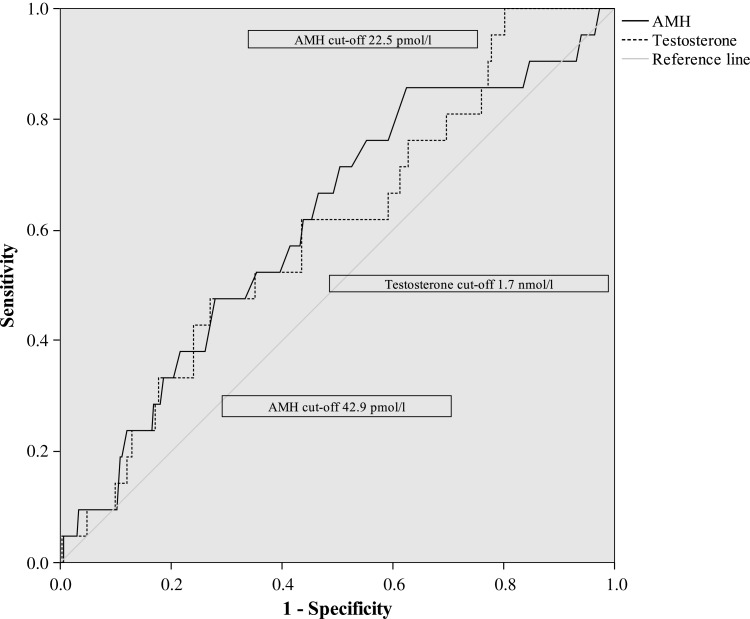
ROC curves of testosterone and AMH for PCOS. Cut-off points with the best sensitivity and specificity used in the analyses are shown in the figure.

Women with PCOS at age 26 (*n* = 21, 5, 3% of the population) had significantly higher AMH levels at age 16 (Table II). The sensitivity and specificity of the serum concentration of AMH was evaluated at age 16 for PCOS at age 26 by using cut-off values according to the ROC-curve (Fig. 3). The sensitivity for PCOS at age 26 with a cut-off value for AMH of 22.5 pmol/l (3.15 ng/ml) was 85.7% and the specificity was 37.5%. When using a cut-off value for AMH of 42.9 pmol/l (6.01 ng/ml), the sensitivity was 33.3% and the specificity was 80.0%. Combination of different AMH and testosterone cut-offs did not significantly improve the accuracy of the test; the best combination was obtained with a cut-off of 22.5 pmol/l for AMH and 1.7 nmol/l for T (ROC-curve analysis, Fig. 3), resulting in a sensitivity of 57.1% and a specificity of 69.9%. An AMH cut-off of 22.5 pmol/l together with a T cut-off of 2.3 nmol/l resulted into a sensitivity of 23.8% and a specificity of 87.6%. An AMH cut-off of 42.9 pmol/l combined with a T cut-off of 1.7 nmol/l resulted into a sensitivity of 23.8% and specificity of 89.5%, whereas the combination with a T cut-off of 2.3 nmol/l resulted into a sensitivity of 4.8% and a specificity of 95.2%.

### AMH and metabolic parameters

In the whole study population, there was a significant but weak correlation between serum AMH levels and BMI at the age of 16 (*r* = 0.124, *P* = 0.013), but the significance disappeared after adjusting for T. At age 16, there was no significant correlation between AMH levels and metabolic indices (WHR, plasma fasting glucose, serum insulin, HOMA-S, hsCRP, serum total cholesterol, HDL-cholesterol, LDL-cholesterol and triglycerides) after adjusting for testosterone and BMI, except weak correlations in the lowest T quartile (WHR: *r* = −0.282, *P* = 0.005, fasting insulin: *r* = 0.290, *P* = 0.004; HOMA-S: *r* = −0.221, *P* = 0.033).

## Discussion

In the present study, we evaluated the relationship between AMH and testosterone, oligo- or amenorrhoea and metabolic and cardiovascular markers, and investigated the value of AMH measurement at the age of 16 in predicting oligo- or amenorrhoea, hirsutism and the diagnosis of PCOS at the age of 26. The results showed that AMH levels associated significantly with T levels and oligo- or amenorrhoea and were a good indicator of oligo- or amenorrhoea in adolescence. A serum AMH levels over 22.5 pmol/l despite its low specificity and therefore poor value as a diagnostic marker, could be used also to identify the girls at risk for PCOS in early adulthood, and allow early prevention by life style counselling.

We previously showed that obesity in adolescence and in adulthood, and also weight gain after adolescence, are associated with self-reported PCOS symptoms in adulthood (Laitinen *et al.*, 2003). Thus, both our previous and present results, and the results from intervention studies treating PCOS (Moran *et al.*, 2011; Teede *et al.*, 2013) suggest that the prevention of obesity and encouragement of physical exercise are important among girls at risk to prevent the syndrome from developing.

Serum AMH levels at age 16 correlated significantly with T levels in adolescence and with self-reported hirsutism scores at age 26, which supports recent findings showing a weak correlation with androgens levels and F&G score (Eilertsen *et al.*, 2012). In line with the present observations, previous studies have shown that increased AMH levels are associated with hyperandrogenism and/or oligo- or amenorrhoea in adolescence (Park *et al.*, 2010a,b) and adulthood (Laven *et al.*, 2004; Li *et al.*, 2011). In our study, however, the correlation with hirsutism was not significant in the whole study population, probably because of the beneficial effect of the OCs on hirsutism.

The correlation between AMH and T levels was the strongest in the highest T quartile, supporting our recent observation that serum T correlates with oligo- or amenorrhoea at age 16 (Pinola *et al.*, 2012). It is probable that the hyperandrogenic girls more often have polycystic ovaries and thereby higher serum AMH levels already in adolescence. Unfortunately, we were not able to verify this finding as ultrasound was not performed in this cohort, but previous studies have shown the association both in healthy and in women with PCOS or with PCO only (Pigny *et al.*, 2003; Hart *et al.*, 2010a,b).

To our knowledge, this is the first longitudinal follow-up study investigating the possible value of AMH as a marker for the development of PCOS later in early adulthood. Interestingly, the highest T quartile was the only one in which AMH at age 16 was significantly higher in women who experienced oligo- or amenorrhoea at age 26 compared with women with a normal menstrual cycle. Based on the present and previous results (Pigny *et al.*, 2003; Hart *et al.*, 2010a,b), we suggest that the girls with irregular cycles and higher testosterone levels at age 16 have a higher antral follicle count and AMH levels and are therefore likely to fulfil the criteria for PCOS later in life. In line with this hypothesis, women with PCOS (either diagnosed or self-reported symptoms according to the questionnaire) at age 26 had higher serum AMH levels in adolescence compared with healthy women and serum AMH levels over 22.5 pmol/l could identify with a sensitivity of 85.7% the adolescent girls at risk for PCOS in early adulthood. The specificity of the test remained weak but improved substantially when using a cut-off of 42.8 pmol/l. Similarly, the results of previous studies have indicated that AMH alone may not be good enough as a single screening tool for PCOS. In the present study, however, the combination of AMH and T did not improve the accuracy of the test, in line with some (Casadei *et al.*, 2013), but not all studies (Eilertsen *et al.*, 2012) in which the power of AMH to diagnose PCOS increased substantially when combined with the other diagnostic criteria of PCOS. The girls with oligo- or amenorrhoea at age 16 and PCOS or hirsutism alone at age 26, however, had higher levels of AMH already at age 16, suggesting that the association between elevated AMH and symptoms of PCOS in adolescence persists also in early adulthood. Using a high enough cut-off value could help to distinguish most of the hyperandrogenic adolescents at risk for PCOS, but a routine use of this test in clinical practice cannot at present be recommended.

We did not find any significant association between AMH and metabolic indices in the whole study population at age 16, which is in agreement with a recent report of a different European population (Anderson *et al.*, 2013). This result indicates that AMH, despite its association with testosterone (Piltonen *et al.*, 2005; Kristensen *et al.*, 2012), is a poor marker of metabolic risks in adolescence. In contrast, in other studies (Nardo *et al.*, 2009; Skalba *et al.*, 2011), AMH associated positively with insulin, HOMA-IR and negatively with HDL, and in one study (Skalba *et al.*, 2011) also positively with total cholesterol and LDL. These studies, however, were performed in older subjects in early adulthood or later, in an age range from 18 to 41. It may be that metabolic risks develop later in life and could not be identified in our study performed in adolescence.

An important pitfall is that AMH assays lack an agreed international standard and therefore the concentrations and cut-off points are method dependent. Importantly, however, in this study all the measurements were made simultaneously using the same AMH assay. Other limitations of this study are that we did not obtain blood samples from the subjects at the age of 26 and that AMH was measured in a subset of the whole cohort. Last, the diagnosis of PCOS at age 26 was based on a questionnaire, but we have previously demonstrated in a similar cohort at age 31 (the Northern Finland Birth Cohort 1966) that self-reported oligo- or amenorrhoea and hirsutism can identify most women with the typical endocrine and metabolic profile of PCOS (Taponen *et al.*, 2003, 2004). Moreover, women with both symptoms fulfilled the Rotterdam criteria for the definition of PCOS (Rotterdam ESHRE/ASRM-Sponsored PCOS consensus workshop group, 2004).

## Conclusion

Serum AMH levels associate significantly with T levels and oligo- or amenorrhoea at age 16 and are a good indicator of oligo- or amenorrhoea in adolescence. Using a high enough cut-off value of AMH to predict which adolescents are likely to develop PCOS in adulthood, despite its low specificity, could help to manage the condition from an early age with a good sensitivity. However, it is not an ideal diagnostic marker and its routine use in clinical practice cannot at present be recommended. Furthermore, AMH is not a good marker for cardiovascular risk factors in adolescence.

## Authors’ roles

P.P.: study design, execution, analysis, manuscript drafting and critical discussion. L.C.M.-P.: study design, execution, manuscript drafting and critical discussion. A.B.: execution and analysis. K.P.: execution and analysis. A.R.: study design and critical discussion. M.-R.J.: study design and critical discussion. S.F.: study design, manuscript drafting and critical discussion. J.S.T.: study design, manuscript drafting and critical discussion. H.L.: study design, manuscript drafting and critical discussion.

## Funding

The study was funded by grants from the Wellcome Trust (089549/Z/09/Z) to H.L., S.F. and M.-R.J. and from Oulu University Hospital Research Funds, Sigrid Juselius Foundation and the Academy of Finland to J.S.T., P.P. and L.C.M.-P. Funding to pay the Open Access publication charges for this article was provided by The Wellcome Trust.

## Conflict of interest

None declared.
